# Epidemiology and Molecular Prevalence of *Toxoplasma gondii* in Cattle Slaughtered in Zahedan and Zabol Districts, South East of Iran

**Published:** 2018

**Authors:** Davood ANVARI, Dariush SAADATI, Reza NABAVI, Majid ALIPOUR ESKANDANI

**Affiliations:** 1. Faculty of Veterinary Medicine, University of Zabol, Zabol, Iran; 2. Dept. of Nutrition and Animal Breeding, Faculty of Veterinary Medicine, University of Zabol, Zabol, Iran; 3. Dept. of Pathobiology, Faculty of Veterinary Medicine, University of Zabol, Zabol, Iran

**Keywords:** *Toxoplasma*, Cattle, PCR, Iran

## Abstract

**Background:**

*Toxoplasma gondii* is an obligate, intracellular parasite which causes the toxoplasmosis in humans and warm-blooded animals. Red meat is an important source for transmission of the infection to humans. This study aimed to determine the prevalence of *Toxoplasma* among imported and indigenous cattle in the Sistan region.

**Methods:**

One hundred samples from slaughtered cattle were collected from two abattoirs of Zabol and Zahedan, South East of Iran in 2015. Each sample was a mixture of three muscle, including tongue, cardiac, and triceps. Additional data of each cattle, including sex, breed, age, indigenous or imported, location of slaughter, management practices, and feeding system were obtained through observations and interviews. Infection by *T. gondii* was determined by PCR method.

**Results:**

The prevalence of *Toxoplasma* in indigenous cattle was 6% and in imported cattle was 26%, and this difference was statistically significant (*P*=0.006). Moreover, the prevalence of *Toxoplasma* was statistically associated with management practices (*P*=0.01) and feeding system (*P*=0.001). However, relationship between the prevalence of *Toxoplasma* with age, breed, sex, and location of slaughter was not statistically significant.

**Conclusion:**

Since the prevalence of *Toxoplasma* among imported cattle is higher than indigenous cattle, so strict supervision for importing livestock from neighboring countries is necessary.

## Introduction

*Toxoplasma gondii* is a protozoan parasite that is spread around the world. Although Toxoplasmosis in human often occur asymptomatically, when the infection is transmitted congenitally, and in immunocompromised patients, it can cause severe symptoms (1). Cat is the definitive host of *T. gondii* and all warm-blooded animals, including humans are intermediate hosts. Definitive host in the early stages of infection excretes several millions of resistant oocysts (2). The oocysts sporulation period is 1–5 d in the natural environmental condition. The infection of intermediate host occurs after ingesting of sporulated oocyst from soil, water or plants (3). Shortly after ingestion, tachyzoites move to be localizing in muscles and nervous system. Finally, the tissue cysts develop including some bradyzoites (3). Humans can be infected either by eating food and water containing infected oocyst or by eating meat containing bradyzoites and tachyzoites (2). Natural infection of cattle with *T. gondii* will not cause clinical symptoms and abortion (4). However, beef plays a special role in epidemiology of toxoplasmosis. Because in some cases beef is consumed as grilled and undercooked, so it is important for the transmission of *Toxoplasma* to humans.

Because the lack of apparent complications on contaminated carcasses, detect of toxoplasmosis in the slaughterhouse during visual inspection is not possible. Various laboratory methods for the detection of toxoplasmosis are used. Molecular methods such as polymerase chain reaction (PCR) assay offer advantages of remarkable sensitivity, high specificity, and speed in diagnosis (5). In the recent years, a large number of imported cattle are slaughtered in Sistan and Baluchestan Province and there is not any information about prevalence of *Toxoplasma* in these animals. Therefore, this study determined the prevalence of *Toxoplasma* among imported and indigenous cattle in the Sistan region.

## Materials and Methods

One hundred cattle carcasses in two abattoirs of Zabol (50 carcasses) and Zahedan (50 carcasses), South East of Iran were sampled in 2015. In any abattoir, half of the samples (25 cases) were indigenous cattle and the others (25 cases) were imported cattle from Pakistan. The samples were taken from each abattoir in three times. In the first, second and third time respectively 16, 16 and 18 carcasses were sampled. Sampling in slaughter line was performed using systematic random sampling method. Additional data of each cattle, including sex, breed, age, indigenous or imported, location of slaughter, management practices, and feeding system were obtained through observations and interviews. Cattle were placed in two categories based on the management practice of the farm to which they belonged. In industrial management, animals are kept at high stocking densities, with large amounts of meat or milk production. These farms are usually far away from residential areas. However, in traditional management, animals are kept in a stable in beside of farmer's house. This farming system is common in rural areas.

### PCR Procedure

Three samples from each carcass, including the tongue, heart, and triceps were taken. The volume of each sample was one cubic centimeter (approximately one gram). The samples were minced by sterile scalpel and the mixture was poured into a sterile Eppendorf tube. Containing each Eppendorf was used following the manufacturer’s instructions of a commercial DNA extraction kit (MBST, Iran) the samples were suspended in 180μL Lysis Buffer and 20 μL proteinase K. Then 360 μL binding buffer was added to each tube and the protocol recommended for tissue samples was followed. All DNA extracts were stored at −20 ºC for use in PCR reaction.

The primers TOX4 (5′CGCTGCAGGGAGGAA-GACGAAAGTTG3′) and TOX5 (5′CGCTGCAGA-CACAGTGCATCTGGATT3′) were selected for PCR assay. These primers amplified a non-coding 529 bp fragment that is repeated 200–300-fold in the genome of *T. gondii* (6). The reaction conditions were as follows: one initial denaturation cycle for 7 min at 94 ºC, 40 cycles of denaturation at 94 ºC for 45 sec, annealing at 55 ºC for 45 sec, and extension at 72 ºC for 45 sec. The procedure was completed by a final cycle extension for 7 min. The positive control was obtained from Parasitology Department, Shahid Beheshti University of medical science. Samples without genomic DNA were included as negative controls.

**Fig. 1:**
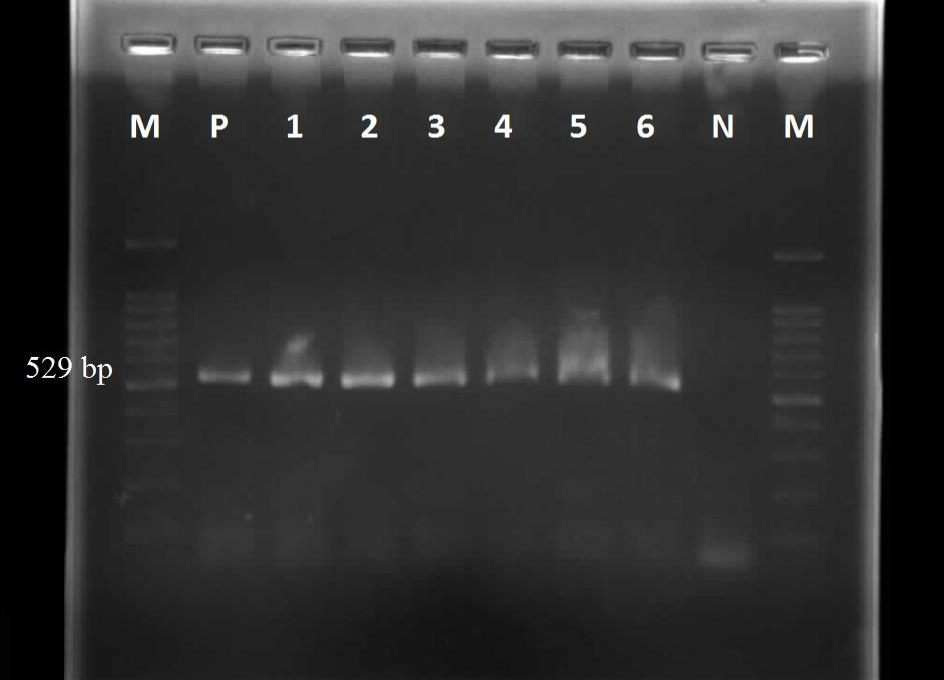
Amplified a non-coding 529 bp fragment for *Toxoplasma gondii. P* is positive control and N represents the negative control, and M is 100 bp marker. Moreover, 1 to 6 is some positive samples.

The PCR products were analyzed on 2% agarose gel and stained using ethidium bromide and visualized in the UV illuminator (Fig. 1).

### Statistical Methods

Association between infection with T. gondii (the dependent variable) and independent variables was investigated using Chi-square test and Fisher exact test. SPSS ver. 18 (Chicago, IL, USA) was used for statistical analysis.

## Results

From 50 samples of indigenous cattle, 3 (6 %) and from 50 samples of imported cattle, 13 (26 %) were shown to be positive by PCR method. The Pearson’s Chi-squared test showed that the observed difference between indigenous cattle and imported cattle is statistically significant. Moreover, traditional livestock and pasture feeding significantly increase the risk of occurrence of toxoplasmosis. However, effects of location of slaughter, breed, sex, and age on prevalence of *T. gondii* are not statistically significant (Table 1).

**Table 1:** The distribution of *Toxoplasma* prevalence based on PCR according to city, location of livestock, type of livestock, feeding, sex, breed, and age

***Category***	***Levels***	***No. of tested animals***	***No. of positive animals***	***Prevalence (%)***	***P-Value***
Location of slaughter	Zabol	50	6	12.0	0.275
	Zahedan	50	10	20.0	
Location of livestock	Indigenous	50	3	6.0	0.006
	Imported	50	13	26.0	
Management practices	Industrial	26	0	0.0	0.01
	Traditional	74	16	21.6	
Feeding	Pasture	30	11	36.7	0.001
	Manual	70	5	7.1	
Sex	Female	3	0	0.0	0.589
	Male	97	16	16.5	
Breed	Holstein	61	13	21.3	0.070
	Sistani	39	3	7.7	
Age	Upper 2 year	85	15	17.6	0.259
	Under 2 year	15	1	6.7	

## Discussion

In this research, the overall prevalence of *Toxoplasma* infection in slaughtered cattle meat was estimated 16.0%. In Ahvaz (Iran), samples of tongue, heart, and muscle were taken from lamb and beef and meat product samples (sausages, hamburgers, and salami) and PCR used for detection of *T. gondii* (7). The overall prevalence of Toxoplasmosis was 14% in lambs, 4% in beef and 0% in meat product samples. The prevalence of *Toxoplasma* in cattle, camels and sheep in the provinces of Isfahan and Chaharmahal and Bakhtiari (Iran) was evaluated using the PCR method (8) that *T. gondii* infections were detected in 0.0%, 6.6% and 17.9% of the samples of cattle (n = 155), camels (n = 122) and sheep (n = 95), respectively. In Lorestan Province (Iran), 174 serums of Cattle were collected from the slaughterhouses, the serum samples were examined via Indirect Immunofluorescence antibody test, and the IgG prevalence rate was 28.73% (9). Among livestock referred to surgery Department of Veterinarian College in Urmia University (Iran), the seroprevalence of *Toxoplasma gondii* in cattle, sheep, and horses were 1.6%, 21.1% and 11.5% (10). In addition, a systematic review and meta-analysis was conducted on the occurrence of toxoplasmosis in cattle in Iran (11). Pooled proportion of toxoplasmosis, among cattle in Iran from 1983 to 2012 was 18.1% (95% CI: 9.9% to 28.2%). seropositive rate of cattle toxoplasmosis in various regions of Iran was between 1.4% and 71.3% in Kerman and Tehran Province (11). Toxoplasmosis was endemic in livestock in the most parts of the country including Sistan region and this is important in terms of the public's health.

Prices of livestock in the eastern neighboring countries are cheaper than Iran. Therefore, in the recent years, a large number of Cattle have been imported from these countries to Sistan and Baluchistan Province. In the present study, the prevalence of *T. gondii* in imported cattle from Pakistan was significantly more than indigenous cattle. In South-West Pakistan, the seroprevalence of *Toxoplasma gondii* infections among 100 cattle was measured by using latex agglutination test (LAT). Positive titers for *Toxoplasma* were detected in 25% of cattle (12). In Punjab Province (Pakistan), 200 serum samples of cattle were examined for the detecting of *T. gondii* by using Latex Agglutination Test (LAT). Overall, 87 cattle (43.5%) were seropositive (13). In private and government-owned farms in Punjab Province (Pakistan), serum samples were taken from 400 cattle. The samples were tested using enzyme-linked immunosorbent assay (ELISA). The overall prevalence of infection was 19.75% (79/400). Moreover, IgG and IgM antibodies were found in 75 cattle (18.75%), and 9 cattle (2.25%) respectively (14). *Toxoplasma* was hyperendemic among Pakistani cattle.

The prevalence of *Toxoplasma* in cattle was associated with feeding system. Cattle graze in the pasture than those fed manually are more likely to become contaminated with sporulated oocysts. In Lorestan Province (Iran), the prevalence of *T. gondii* in cattle was lower than sheep and goats (29% vs 53% and 52%). Cattle were often fed manually, but sheep and goats graze in pastures (9).

The prevalence of *Toxoplasma* in industrial livestock was significantly less than traditional livestock and stray cats roaming mostly on traditional farms. They can contaminate water and forage in their feces. A significant association was found between prevalence of *Toxoplasma* and exposure of cattle to cats. Including a study on risk factors of toxoplasmosis in large ruminants showed that presence of cats in the surrounding areas of cattle is an important risk factor for infection of cattle (14). In addition, in Brazil, seroprevalence of *T. gondii* in cattle was depended on number of cats in farm, contact of cats with cattle and contact of cat with drinking water (15). Besides, Proper hygiene in industrial farms is due to the less occurrence of toxoplasmosis in this livestock. In addition, in this study, all cattle reared industrially, were feeding manually, and the low prevalence of *Toxoplasma* in such industrial group is related to feeding pattern. Similar findings in sheep have also been reported in Zimbabwe. Toxoplasmosis in Sheep from commercial farms was significantly lower than that of Sheep from the communal grazing system (16).

In the present study, the prevalence of infection was not statistically associated with location of slaughter, age, breed, and gender of cattle. The prevalence of this parasite in female cattle was statistically more than male animals (17), as well as female animals, were more susceptible than males for toxoplasmosis (18). However, in Tabriz (Iran), antibody to *Toxoplasma* in male cattle was significantly more than females (19).

## Conclusion

The prevalence of *Toxoplasma* gondii in the meat of imported cattle from Pakistan is higher than indigenous livestock. Since the *Toxoplasma* endangers human health, meat of imported cattle could be freeze before offering to market for decreasing of occurrence of toxoplasmosis in humans. Moreover, strict supervision for importing livestock from neighboring countries is necessary.
